# Microwave detection and quantification of water hidden in and on building materials: implications for healthy buildings and microbiome studies

**DOI:** 10.1186/s12879-019-3720-1

**Published:** 2019-01-18

**Authors:** Andrew Horsley, David S. Thaler

**Affiliations:** 1 Department of Physics, University of Basel, Klingelbergstrasse 82, CH-4056 Basel, Switzerland; 2 Research School of Physics and Engineering, The Australian National University, Mills Rd., ACT 2601, Canberra, Australia; 3 Biozentrum, University of Basel, Klingelbergstrasse 50/70, CH-4056 Basel, Switzerland

**Keywords:** Aquametry, Dampness, Humidity, Microbiome, Microwave, Moisture, Mold, Sick-building-syndrome

## Abstract

**Background:**

Excess water in all its forms (moisture, dampness, hidden water) in buildings negatively impacts occupant health but is hard to reliably detect and quantify. Recent advances in through-wall imaging recommend microwaves as a tool with a high potential to noninvasively detect and quantify water throughout buildings.

**Methods:**

Microwaves in both transmission and reflection (radar) modes were used to perform a simple demonstration of the detection of water both on and hidden within building materials.

**Results:**

We used both transmission and reflection modes to detect as little as 1 mL of water between two 7 cm thicknesses of concrete. The reflection mode was also used to detect 1 mL of water on a metal surface. We observed oscillations in transmitted and reflected microwave amplitude as a function of microwave wavelength and water layer thickness, which we attribute to thin-film interference effects.

**Conclusions:**

Improving the detection of water in buildings could help design, maintenance, and remediation become more efficient and effective and perhaps increase the value of microbiome sequence data. Microwave characterization of all forms of water throughout buildings is possible; its practical development would require new collaborations among microwave physicists or engineers, architects, building engineers, remediation practitioners, epidemiologists, and microbiologists.

## Background

Visible signs of dampness and mold in buildings are epidemiologically associated with adverse health outcomes for occupants [1–3]. However, there is a quantitatively large variance in studies of such health outcomes [4, 5], and there is a need for deeper understanding of these associations. In addition to factors such as genetic and experiential differences among occupants, a key confounding factor is the limited availability of data on the location and amount of water present. Moreover, information on the presence of water is critical for implementing any remedial action. The most added-value from improved water detection would be expected from methods that are noninvasive, quantifiable, spatially resolved, and able to detect hidden water. The capability to passively monitor over long timescales is also valuable, in order to monitor for water that may only appear sporadically.

Current non-destructive technologies for the measurement of water in buildings have significant limitations, especially in the detection of hidden water [6–8]. Unaided visual inspection is widely used to assess outer surfaces for signs of water damage or mold, with improved sensitivity sometimes offered by infrared imaging of surface temperature [9]. Infrared imaging relies on detecting the temperature differentials that can form between wet areas, which are often relatively cool, and the surrounding dry areas [10]. To the best of our knowledge, the reviewed literature has not yet critically examined infrared detection of hidden water with regard to sensitivity, quantitative reliability, confounding factors, and how deeply into building materials infrared-based detection can penetrate.

The ability of microwaves to penetrate through walls make them an attractive solution for the detection of hidden water. Microwave aquametry [11] is already used to measure moisture during the preparation of building materials such as wood [12, 13] and concrete [14, 15], as well as in a range of other materials such as soils [16], seeds [17], cheese [18] and textiles [19]. Moisture monitoring within building walls has been performed with qualified success by measuring microwave transmission between probes drilled into the wall [20], however this partially-destructive technique has not been broadly adopted.

The present paper makes no claim to have developed a microwave technology that works in a practical way to detect hidden water in buildings. However, we provide a simple demonstration of the detection of small volumes of water in and around common building materials. Interdisciplinary collaboration and engineering efforts will be required to turn this demonstration into a practical device or application. Practical development will be further considered in the discussion.

## Methods

**Fig. 1:**
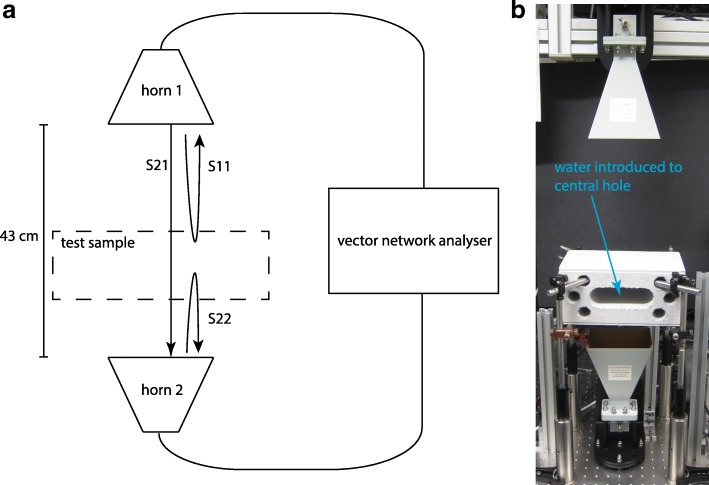
Detector setup. **a** Schematic of the setup, showing the vertically separated microwave horns, driven by a vector network analyser. The microwave transmission (S21) and reflection (S11, S22) S-parameters are indicated with arrows. **b** Photo of the setup, with the concrete brick as test sample. For scale, the screw holes in the table are 25 mm apart

We used a simple setup, consisting of two microwave horns (A-info, LB-OH-159-15-C-SF) connected to a vector network analyzer (Agilent, PNA N5222A), as shown in Fig. 1(a). This allowed us to measure the microwave reflection and transmission through test samples placed between the horns, as a function of microwave frequency.

The reflection and transmission are measured as S-parameters. As indicated in Fig. 1(a), S11 (S22) measures reflection of a signal sent from horn 1 (2), and S21 measures transmission from horn 1, through the test sample, to horn 2. Starting with a dry test sample, we used a pipette add water in 1 mL steps and monitored the resulting change in S-parameters, making measurements within a few seconds of each step. For an S-parameter S_γ_ (γ = 11, 22, 21), we define the change in reflection or transmission due to the added water as ΔS_γ_ = S_γ_ - S_γ0_, where S_γ0_ is the S-parameter measured without any water present. The 4–8 GHz bandwidth of our measurements was chosen to match the bandwidth of the available microwave horns, and the network analyser output power was 0 dBm (1 mW).

## Results

**Fig. 2:**
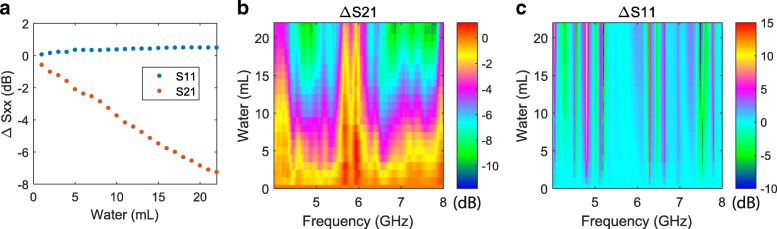
S-parameter measurements adding water to a concrete brick. **a** Microwave transmission (S21) and reflection (S11) averaged over the 4–8 GHz measurement bandwidth. **b** S21 and (**c**) S11 as a function of microwave frequency

To demonstrate the suitability of microwaves for detecting water in inaccessible spaces, such as inside walls, we used a hollow concrete brick, shown in Fig. 1(b), with 7 cm of concrete above and below the central hole. The brick was dried in air for 1 week before the measurement. Figure 2 shows the changes in transmitted and reflected microwave signals as we added water with a pipette, creating a free-standing water layer in the hollow centre of the brick. We detect water volumes as small as 1 mL, and see a strong increase in absorption with increasing water volume. There is little change in the reflected signal with water volume, however we do see oscillations in reflectivity (and to a lesser extent absorption) as a function of microwave frequency. We attribute this to interference between reflections from the water-brick and water-air surfaces, which depends on the ratio of microwave wavelength to water layer thickness (see discussion below). We did not see oscillations as a function of water volume in this experiment. We interpret this lack of change with water volume as follows: the area covered by the water layer in the brick increased with volume, however the thickness (roughly 1–2 mm) remained constant. Absorption of water into the brick occurred over tens of minutes, and was negligible over the 7 min measuring time. Water loss due to evaporation, which is strongly dependent on airflow velocity [21] can also be assumed to be negligible within the confines of both the hollow brick and our lab.

**Fig. 3:**
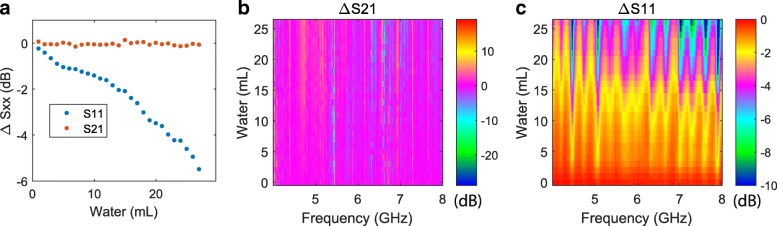
S-parameter measurements adding water to the surface of an aluminium sheet. **a** Microwave transmission (S21) and reflection (S11) averaged over the 4–8 GHz measurement bandwidth. **b** S21 and (**c**) S11 as a function of microwave frequency

Metallic objects in a building, such as pipes, will block microwave transmission. We show that water on a metallic surface can be detected through its influence on the reflected microwave signal. We used a 5 mm thick aluminium sheet as test sample, and created a free-standing water layer directly on top. As transmission through the aluminium was essentially zero, Fig. 3(a + b) show minimal variation in transmitted signal with water volume. However, Fig. 3(a + c) do show a strong decrease in reflection (S11) with water volume, and we again detect volumes down to 1 mL. This change in reflection signal, which was not seen in Fig. 2, is due to the water blocking the signal from the aluminium surface. We again see oscillations in reflection as a function of frequency but not water volume, due to interference between the water-aluminium and water-air interfaces, and the fact that increasing water volume did not change the water layer thickness.

**Fig. 4:**
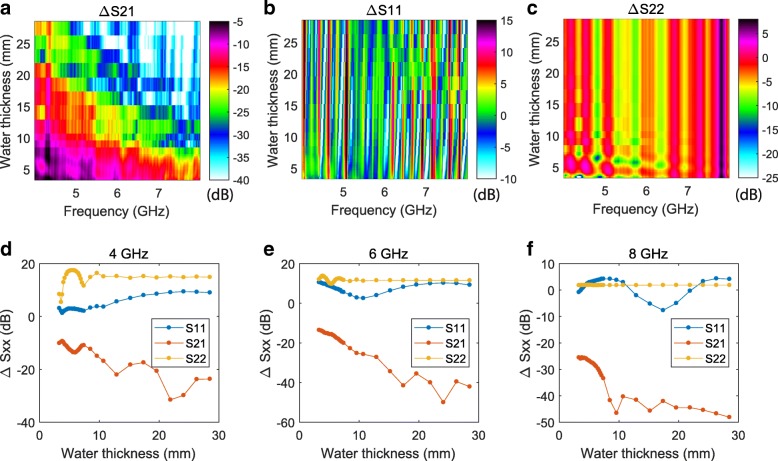
S-parameter measurements adding water to a pyrex dish. **a** Microwave transmission (S21), (**b**) reflection (S11) and (**c**) reflection (S22) as a function of microwave frequency. **d**-**f** S-parameters averaged over 0.1 GHz frequency bands respectively starting at 5, 6, and 7 GHz

To demonstrate the effect of water layer thickness, we used a Pyrex container as test sample, which ensured that the water layer thickness increased approximately linearly with water volume. Figure 4 shows the changes in microwave absorption and reflection, where we can see S-parameter oscillations as a function of both frequency and water thickness. Figure 4(d-f) show line cuts for different frequencies, where we can see that the oscillation period with water thickness is different for each S-parameter, and varies with microwave frequency.

**Fig. 5:**
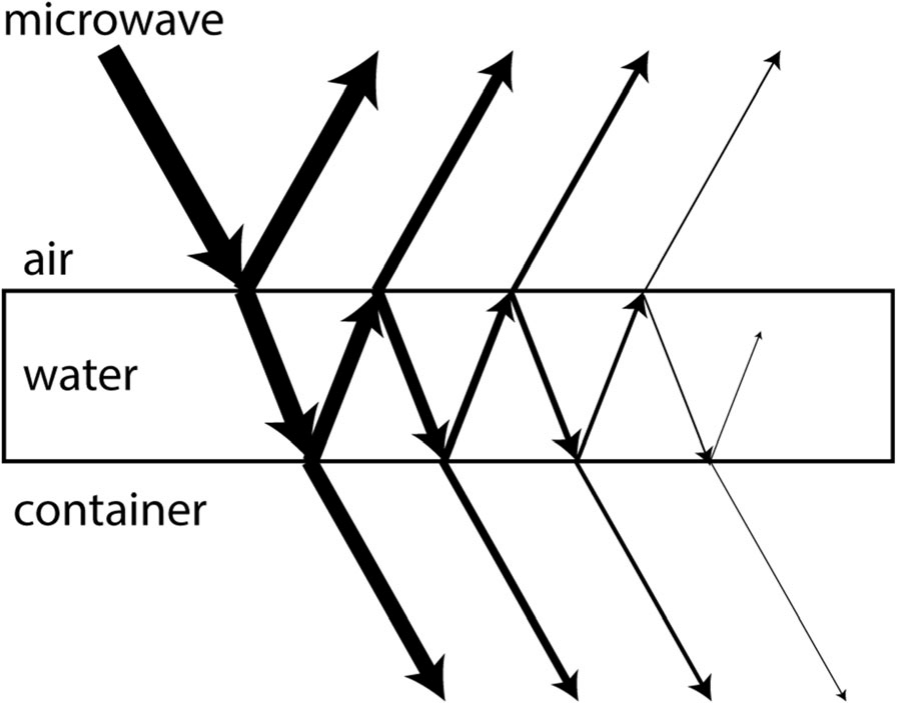
Thin-film interference: the incident microwave will be partially transmitted and partially reflected at each boundary (air-water or water-container) it encounters. The multiple paths taken by the microwave can interfere with one-another, resulting in oscillations in the net transmission and reflection as a function of parameters such as water layer thickness and microwave wavelength

We can understand the S-parameter oscillations by considering microwave interference effects in a thin dielectric film, as described in classical optics [22]. The incident microwave undergoes multiple transmission and reflection events at the air-water and water-container boundaries (see Fig. 5), producing waves which interfere with one-another. In the most simple picture, the net reflection and transmission coefficients oscillate sinusoidally with a frequency proportional to *nd* cos(*θ*)/*λ*, where *n* is the complex refractive index of water, *d* is the water thickness, *θ* is the microwave angle of incidence, and *λ* is the microwave wavelength. This qualitatively explains the observed S-parameter oscillations as a function of microwave frequency (∝1/*λ*) and water thickness, and also the faster oscillations as a function of water thickness for higher microwave frequencies, where the *d*/*λ* ratio is larger. The amplitude of the S-parameter oscillations as a function of water thickness decays faster at higher microwave frequencies (Fig. 4c), which is due to the absorptive component of the refractive index increasing with microwave frequency [23]. Accurate modelling of the quantitative features of the S-parameter oscillations, such as how the oscillation frequency is different for S21, S11 and S22, and for different measurement setups, is beyond the scope of this work. These features may be explained through explicit consideration of factors such as microwave attenuation in the water, integration over a range of *θ* for each microwave horn, and the material-dependence of reflection and transmission at the various water-(wet/dry) concrete, water-aluminium, and water-Pyrex boundaries. In future setups, these factors may be best accounted for by performing 3D holographic reconstruction of spatially resolved measurements [24].

## Discussion

### Microwaves and water

The microwave characteristics of building materials differ from water in absorption, reflection, and refraction. Common building materials, such as the concrete block used in this study, are almost transparent to microwaves. Communications networks (Wifi, cell phones, etc.) exploit this fact on a daily basis. On the contrary, water is strongly absorptive at microwave frequencies. The difference between these materials with respect to microwave absorption is inverted with respect to visible light, allowing microwaves to detect water where visible light cannot penetrate. Metals, such as steel pipes and beams, will block microwave transmission, but metallic surfaces are highly reflective at microwave frequencies. Water on the surface of metal can be detected through its influence on reflected microwave signals.

### Integrating water detection with other building research that uses microwaves

Microwave imaging techniques that can be applied indoors and through-walls [24–29] are under development for applications including emergency response, security, and radio-tag tracking for an internet-of-things. The development of hidden water imaging can benefit greatly from the technologies developed for these applications, whose technological requirements are close to those of hidden water detection and quantification. Imaging techniques may soon be extended to perform holographic imaging of entire buildings, whilst hardware requirements may be minimized by taking advantage of the background radiation from wifi routers [24]. The spatial resolution of microwave tomography is currently limited by the microwave wavelength, i.e. approximately the 1-10 cm range. Resolution might be improved further by other means, e.g. through incorporating nearfield detection, perhaps in conjunction with non-canonical detectors [30–32].

### Moisture and microbiomes

Detection and localization of moisture in buildings can yield an understanding of the presence and activity of microbes and microbial products, an important factor in understanding the impact of the building environment on occupant health [33–35]. Hidden spaces in buildings are difficult to access yet their moisture levels may be critical for understanding the building microbiome.

The presence of water is essential for all living metabolism and growth, and there is a strong correlation of measured dampness with visible signs and/or odors of microbial growth in buildings [8]. A focus on the ability to detect small amounts of water in the built environment (the lower limit of water activity required for microbial metabolism is subject to ongoing research [36–38]) would facilitate earlier discovery of conditions enabling microbial growth, with the detection of hidden water, i.e. water which is not present on easily-accessed surfaces, of particular importance. Current and potential problems could then be detected before affecting the health of occupants [39, 40], and building remediation would be simplified by the reduction in building damage due to earlier and more reliable detection. Information on the presence of water could also improve the value of analyses based on DNA [41, 42] or Volatile Organic Compounds (VOCs) [43–45], because locations with water often correlate with metabolically active microbes. On the other hand, the consequences of moisture on the health of occupants need not always be directly dependent on microbes. Emission and adsorption of VOCs [46, 47] and inorganic but health-relevant radon gas [48–50] are also sensitive to moisture and humidity.

A building’s microbiome comes from three distinct sources [51]: a) dispersal from occupants; b) the outside environment; and c) microbial growth in the building itself. Building-associated illness has been linked to type c [39, 40]. However, a comparison of studies concerning the human health effects of microbial exposure in buildings reveals an apparent paradox: some studies indicate detrimental health effects while others correlate benefits with microbial exposure [52]. Better data concerning current and historical moisture conditions in buildings, especially at microbiome assay sites, may help clarify and, perhaps, resolve this apparent paradox.

In the last 10 years, there has been spectacular progress in techniques for microbiome characterization in buildings, in particular through Next Generation Sequencing (NGS) of DNA [41] and also VOC sensing [43–45]. However, these sophisticated methods do not fully address the need to locate and characterize microbial growth and metabolism occurring in building structures (for an exemplary exception to this critique see Adams et al. 2017 [42]). For example, a given microbiome DNA sequence should be interpreted differently depending on whether the corresponding microbe was metabolically active, but DNA sequences alone do not provide information on whether identified sequences come from organisms that were metabolically active, quiescent, or dead. DNA sequencing can be supported by methods that discriminate microbial viability at the time of sampling [53] and the intact nature [54] of target DNA, whilst certain VOCs are consequent to microbial metabolism [44]. However, these approaches are not always sensitive and are not likely to be robust across the range of microbial and environmental diversity. Enthusiasm for NGS may have skewed microbial ecology approaches in the building research community away from classical microbiology, which has for a long time been appreciative of the key role of hidden water in building microbiology [55, 56].

The difficulty of making sequence-based microbiome research relevant to practical problems was addressed in the meeting summary for the International Society for Indoor Air Quality and climate symposium at Healthy Buildings 2015-Europe [57]: “There was general consensus that the applied microbiology developments emerging in this research community —first and foremost, DNA recovery methodology and in particular, next-generation sequencing —have had notable impacts as judged by common academic metrics; however, these advances have not successfully translated into paths which are available for practitioners to apply such methods or interpret these results with confidence in the field.”

The needs of building remediation practitioners were clearly articulated [58]: “Some buildings are obviously in need of remediation. Some buildings are obviously fine. However, there are a large number of intermediate buildings. Residents may express the concern that a building is making them ill but there is no visible problem. We may have many buildings with small or intermediate signs of problem dampness and/or microbial growth but we have no objective way to rank or prioritize them in terms of the necessity for, or order in which to undertake remediation. Furthermore, after remediation activity in a building has been completed, we do not have an objective way to prove that it worked.” The practitioner then asked if microbiome analysis could provide what he and other practitioners need. The consensus answer of the building microbiome and building science researchers at the meeting was that it could not. A US National Academy of Science 2017 study on microbiomes in the built environment also addresses this need as a research priority: “Critical guidance is lacking on when to initiate interventions for damp buildings and on how to gauge the success of these interventions.” [56]**.**

### The need for an interdisciplinary research and development program

The work reported here is an interdisciplinary collaboration of a physicist (AH) with expertise in microwaves and a microbiologist (DST) who has had substantive interactions with the healthy building community. Further development would be most efficient and also exciting through the active collaboration of the healthy building community and microbiologists, alongside microwave engineers and physicists. Health benefits are anticipated to follow from more accurate and objective criteria for assessing building engineering, remediation and design options.

Recent advances in microwave imaging techniques, demonstrated in studies mapping the positions and movement of people and objects in rooms [24–26, 28] and through walls [27, 29], provide a potential building-scale hidden water imaging solution. Imaging of water inside thermal insulating building materials has already been shown using holographical radar techniques [59]. Microwave illumination could be provided by a user-controlled source, or imaging could be performed using the substantial background microwave field associated with cell phone networks and Wifi routers in the contemporary built environment [24].

## Conclusions

We propose that improved detection and quantification of hidden water in buildings would enable more efficient and effective building design and remediation leading to improved public health. Better data on the state of water in all its forms over time might improve the relevance of microbiome analysis to the health of building occupants. This paper includes a demonstration that microwave sensing offers one approach for the problem of detecting hidden water in the built environment. The most effective realization would be best accomplished via an interdisciplinary research program including the healthy building disciplines, microwave engineering or physics, and microbiology as related to epidemiology.

## Abbreviations

NGS: Next Generation Sequencing
S11: reflection coefficient
S21: transmission coefficient
S22: reflection coefficient
S-parameter: Scattering-parameter
VOC: Volatile Organic Compound
